# Spontaneous Eye Blink Rate During the Working Memory Delay Period Predicts Task Accuracy

**DOI:** 10.3389/fpsyg.2022.788231

**Published:** 2022-02-15

**Authors:** Jefferson Ortega, Chelsea Reichert Plaska, Bernard A. Gomes, Timothy M. Ellmore

**Affiliations:** ^1^Department of Psychology, The City College of the City University of New York, New York, NY, United States; ^2^Behavioral and Cognitive Neuroscience Program, The Graduate Center of the City University of New York, New York, NY, United States; ^3^Department of Neurosurgery, Cedars-Sinai Medical Center, Los Angeles, CA, United States

**Keywords:** spontaneous eye blink rate, working memory, delay period, dopamine, attention

## Abstract

Spontaneous eye blink rate (sEBR) has been linked to attention and memory, specifically working memory (WM). sEBR is also related to striatal dopamine (DA) activity with schizophrenia and Parkinson’s disease showing increases and decreases, respectively, in sEBR. A weakness of past studies of sEBR and WM is that correlations have been reported using blink rates taken at baseline either before or after performance of the tasks used to assess WM. The goal of the present study was to understand how fluctuations in sEBR during different phases of a visual WM task predict task accuracy. In two experiments, with recordings of sEBR collected inside and outside of a magnetic resonance imaging bore, we observed sEBR to be positively correlated with WM task accuracy during the WM delay period. We also found task-related modulation of sEBR, including higher sEBR during the delay period compared to rest, and lower sEBR during task phases (e.g., stimulus encoding) that place demands on visual attention. These results provide further evidence that sEBR could be an important predictor of WM task performance with the changes during the delay period suggesting a role in WM maintenance. The relationship of sEBR to DA activity and WM maintenance is discussed.

## Introduction

The healthy human blinks around 15–20 times per minute (Tsubota et al., 1996), however the precorneal tear film, which lubricates the eye, only begins drying up approximately 25 s after a blink ends (Norn, 1969). This suggests that we blink more often than needed to maintain a lubricated precorneal tear film. Previous research has found task-related modulation of spontaneous eye blink rate (sEBR), which indicates that blinking could be reflective of cognitive factors (Siegle et al., 2008; Oh et al., 2012). For example, reading is accompanied by low levels of sEBR, while high levels of sEBR have been reported during conversation (Bentivoglio et al., 1997). More recent studies have found that sEBR correlates with attentional load and fatigue (Maffei and Angrilli, 2018), attentional control (Colzato et al., 2009; Unsworth et al., 2019a), can track working memory updating and gating (Rac-Lubashevsky et al., 2017), and can predict differences in exploration during reinforcement learning (Van Slooten et al., 2019). In addition, a growing body of literature continues to provide evidence supporting sEBR as an effective measure of striatal dopamine (DA) activity (Jongkees and Colzato, 2016). However, whether sEBR does indeed reflect DA activity is still debated today (Dang et al., 2017; Sescousse et al., 2018).

The connection between sEBR and DA first came from observations of neurological and psychiatric disorders that found decreased sEBR in patients with Parkinson’s (Hall, 1945; Reddy et al., 2013), a neurodegenerative disorder that affects the dopaminergic system in the brain, causing symptoms like rigidity (Dauer and Przedborski, 2003). Schizophrenia has also been suggested to provide evidence for a connection between sEBR and DA due to excessive DA activity in the striatum (Howes et al., 2015) and increased sEBR in schizophrenia patients (Adamson, 1995; Swarztrauber and Fujikawa, 1998). Additionally, sEBR and DA has previously been investigated in pharmacological studies, which have observed an increase in sEBR after administration of DA agonists, while DA antagonists decreased sEBR in primates (Elsworth et al., 1991; Jutkiewicz and Bergman, 2004). In one study, researchers found sEBR was correlated with dopamine levels specifically in the caudate nucleus in monkeys, suggesting that DA could regulate blink rate (Taylor et al., 1999). This is further supported by another study that found sEBR to be closely related to *in vivo* and positron emission tomography measures of striatal D2 receptor density in the ventral striatum and caudate nucleus of adult male vervet monkeys (Groman et al., 2014). These findings provide valuable evidence for sEBR being a viable measure of striatal DA activity and have led to many researchers to adopt sEBR as a measure of DA activity. Moreover, sEBR is an easy-to-record physiological measure that is non-invasive and affordable.

One cognitive process of interest, that is also closely related to DA activity, is working memory (WM) which is the process of actively holding information online and manipulating it to meet task demands (Baddeley, 1992). Prior research has found substantial evidence that demonstrates the importance of dopaminergic neurotransmission and the role of the prefrontal cortex during WM function (Fuster and Alexander, 1971; Funahashi et al., 1989; Courtney et al., 1998; Wager and Smith, 2003; Cools and Robbins, 2004), especially during WM maintenance (Fuster and Alexander, 1971; Funahashi et al., 1989; Constantinidis et al., 2018). Specifically, human studies investigating DA in WM tasks have found both caudate dopamine activity during WM maintenance and DA synthesis capacity to be positively correlated with WM capacity, a measure of the amount of information that can be held in WM (Cools et al., 2008; Landau et al., 2009). Though it is widely accepted that the PFC plays an important role in WM function (Roberts et al., 1998), many researchers still debate the PFC’s role in WM (Seamans and Yang, 2004). One model that attempts to elucidate the PFC’s role in WM function is the prefrontal cortex basal ganglia WM model (PBWM; Frank et al., 2001; Hazy et al., 2006). PBWN is a computational neural network model that suggests that WM requires robust maintenance and rapid selective updating. This model states that the frontal cortex facilitates robust, active maintenance through recurrent excitation in frontal neurons, while the basal ganglia orchestrates a gating mechanism that controls the flow of information into WM (Frank et al., 2001). Previous research has pointed toward DA being important for this sustained firing activity in the PFC during WM maintenance (Sawaguchi, 2001; Durstewitz and Seamans, 2008; De Frias et al., 2010). The relationship between DA and WM performance is believed to follow an inverted U-shape, in which too little or too much dopamine impairs performance, as seen in psychopharmacological studies (Stewart and Plenz, 2006). In one study, the effects of administered dopaminergic drugs on PFC function depended on baseline levels of performance, whereas administration of bromocriptine, a dopamine agonist, impaired performance for individuals with higher working memory abilities while improving performance for individuals with lower working memory abilities (Kimberg et al., 1997).

Although sEBR has been used in prior research to investigate cognitive functions like WM, many of these studies relied on baseline levels of sEBR to investigate these relationships (Tharp and Pickering, 2011; Zhang et al., 2015; Unsworth et al., 2019b). Few studies have investigated the relationship between phasic sEBR during a WM task. Phasic sEBR refers to the measuring of sEBR in response to stimulus conditions while tonic sEBR refers to baseline levels of blinking (Bacher and Allen, 2009). To the best of our knowledge, only one other study has examined sEBR as a function of the different task phases (e.g., stimulus encoding, maintenance during the delay period, and stimulus probe periods) of a WM task (Bacher et al., 2017). Bacher et al. (2017) found modulation of sEBR across these different phases are developed in infants as young as 10 months, indicating that sEBR can reflect dopamine function in early human development. They also observed higher sEBR during the Hide (delay) phase of the task in relation to the Reveal phase, which is when the experimenter revealed the toy’s location to the child. This modulation of sEBR was suggested to reflect the engagement of cognitive resources that have become available during the Hide phase and transiently elevated DA activity that is needed to update and maintain mental representations (Bacher et al., 2017).

**Figure 1:**
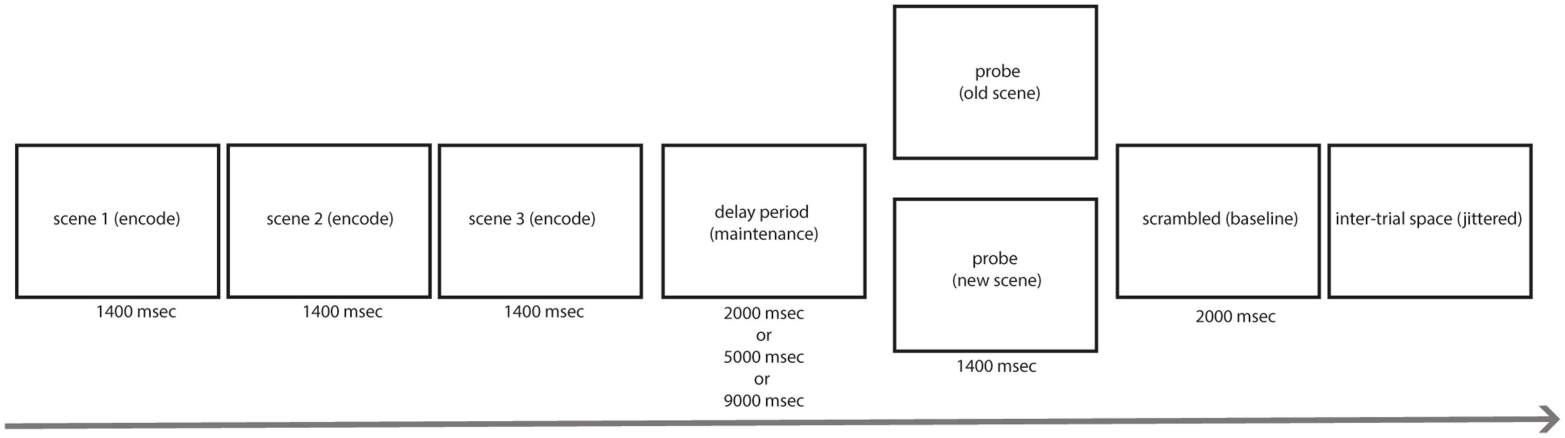
Task design. Each trial began with an encoding period where three novel complex scenes were presented for 1,400 ms each. The encoding period was followed by a delay period where a fixation cross was presented on a gray background for a varied amount of time (2 s, 5 s, or 9 s). After the delay period, the probe was presented for 1,400 ms and participants had to identify whether the image was a new image or one of the previously presented images with a button press. After the probe, a scrambled image was presented for 2,000 ms which indicated the end of the trial followed by jittered blank space before the start of the next trial.

The goal of the current study was to investigate how fluctuations in sEBR during different phases of a Sternberg visual WM task (Figure 1) relate to performance, and how sEBR fluctuations change across task demands. First, we hypothesized that sEBR during the WM Delay period, when stimuli are being maintained, would be positively correlated with task performance and that there would be a non-linear relationship such that low and high sEBR would correlate with worse performance. Second, we hypothesized differences in sEBR across phases of the WM task with differences between phasic sEBR during the WM delay and tonic sEBR during non-task rest periods.

## Materials and Methods

### Participants

The experiments were conducted under a protocol approved by the Institutional Review Board of the City University of New York Human Research Protection Program (CUNY HRPP IRB). All methods were carried out in accordance with the relevant guidelines and regulations of the CUNY HRPP IRB committee. All participants were recruited either by flyers posted throughout the City College of New York campus or by web postings on the City College of New York SONA online experimental scheduling system. All participants had normal or corrected-to-normal vision with no reported neurological or psychiatric disorders. Participants were either compensated $15 per hour or received one psychology course credit per hour of participation in the study. Written informed consent was obtained from all participants in the study.

Participants selected for Experiment 1 and Experiment 2 were part of a larger study. Nineteen healthy participants (8 males; *M* = 23.79; *SD* = 7.72) were recruited for Experiment 1. In Experiment 1, sEBR was measured inside a 3 tesla Siemens Prisma MRI scanner. In Experiment 2, sEBR was recorded in a sound attenuated EEG booth during acquisition of EEG data while participants sat upright. Fifty-three healthy participants (29 males; *M* = 23.58; *SD* = 5.79) were recruited for Experiment 2. Three participants were removed from Experiment 1 including one participant who was removed for noisy data and two who were removed for task performance below or close to chance. A total of 19 participants were removed from Experiment 2 for multiple reasons including 11 participants who were removed due to bad EOG channel quality, four participants who were removed because of a stimulus marker malfunction, three participants who were removed due to outlier detection, and two who were removed for failing to adhere to the protocol. The final sample for the analysis in Experiment 1 was 16 subjects, and for Experiment 2 was 34 subjects.

### Task and Procedure

Prior to the start of the task, participants completed a 5-min Rest period which consisted of staring at a black fixation cross that was shown on a gray background. Participants completed another 5-min Rest period after completing three runs of the task. This fixation cross was also used during the delay period of the task. Participants completed three runs, each run containing 54 trials, of a modified version of the Sternberg WM task (Sternberg, 1966). Naturalistic scenes were used as stimuli and were sampled from the SUN database (Xiao et al., 2010). The task consisted of a stimulus encoding period, delay period, probe period, and post-probe scrambled stimulus period (which served as a visual baseline and to signal end of trial). During the encoding period, participants were shown three subsequent novel scenes for 1400 ms each. During the delay period, a black fixation cross was shown on a gray background for varied lengths (either 2, 5, or 9 s long). The delay period duration was randomized from trial to trial to engage subjects’ attention consistently across trials because they could not predict when the delay period would end. Each three runs of the task had 18 trials of each delay duration with order of presentation randomized. The probe was presented for 1400 ms after the delay period and consisted of a new image (one that has not been presented yet) or an old image (one that was shown during encoding). The chance of receiving a new probe was 50%. Participants indicated whether the image presented was either a new or an old image with a button press. After the probe, a Fourier phase-scrambled scene was shown for 2000 ms, indicating the end of the current trial followed by a jittered period of blank screen.

### sEBR Recording

Participants were not given instructions about when to blink during the experiment. Previous studies have found blink rate to be stable between 10 am and 5 pm (Barbato et al., 2000; Doughty and Naase, 2006). For both Experiment 1 and Experiment 2, sEBR was recorded between 10:00 am and 3:00 pm. During Experiment 1, eye blinks were recorded inside a three tesla Siemens Prisma MRI scanner using an MRI compatible EyeLink 1,000 Eye Tracker (SR Research) and was recorded at 500 Hz. In Experiment 2, eye blinks were recorded using an electrooculogram (EOG) that was recorded during 64-channel scalp electroencephalogram using a Brain Products cap and active electrode recording system. EOGs were placed above the left eye and below the right eye to track blinking. Blink detection was performed using MNE Python *via* the function “*find_eog_events*” (Gramfort et al., 2013). Blink epochs were evaluated for each run of the task for all participants. Runs with blink epochs which did not resemble the standard blink shape were removed from the analysis. Only participants with 2 or more runs of good eye-tracking data were used in the analysis. The first 2 s of all delay periods were used in the analysis. In Experiment 1 and 2, sEBR was computed by dividing the total number of blinks by the total period duration for any given phase resulting in units of blinks per minute:


$$sEBR=\frac{total\;blinks}{total\;period\;duration}$$

### Statistical Analysis

Statistical analyses were computed using JASP Version 0.16. sEBR and task accuracy data were checked for outliers prior to analysis and were removed. Because the relationship between sEBR and WM performance is believed to follow an “inverted U-shape,” we did not consider Pearson’s r the optimal measure for this analysis because it is limited to evaluating only a linear relationship between two variables. Instead, we computed Spearman’s rho, which can describe monotonic functions, whereas the value of one variable changes the other variable changes but not necessarily at a constant rate. We also used polynomial regression analysis, which is more appropriate for quantifying non-linear associations. Specifically, we investigated the non-linear relationship between task accuracy and sEBR during the Delay period of the task for both Experiment 1 and Experiment 2 using a second order polynomial regression model. Unidimensional reliability analyses were computed using task accuracy and sEBR as input variables and Cronbach’s α as the frequentist scale reliability statistic. Post-hoc statistical power calculations were computed for each experiment and the combined samples of both experiments with G^*^Power Version 3.1.9.6 using the correlation between sEBR during the Delay period and task accuracy. Parameters included the Exact test family, Correlation: Bivariate normal model with an α error probability of 0.05, the sample size, and the correlation coefficient as the effect size.

## Results

### Experiment 1

**Figure 2:**
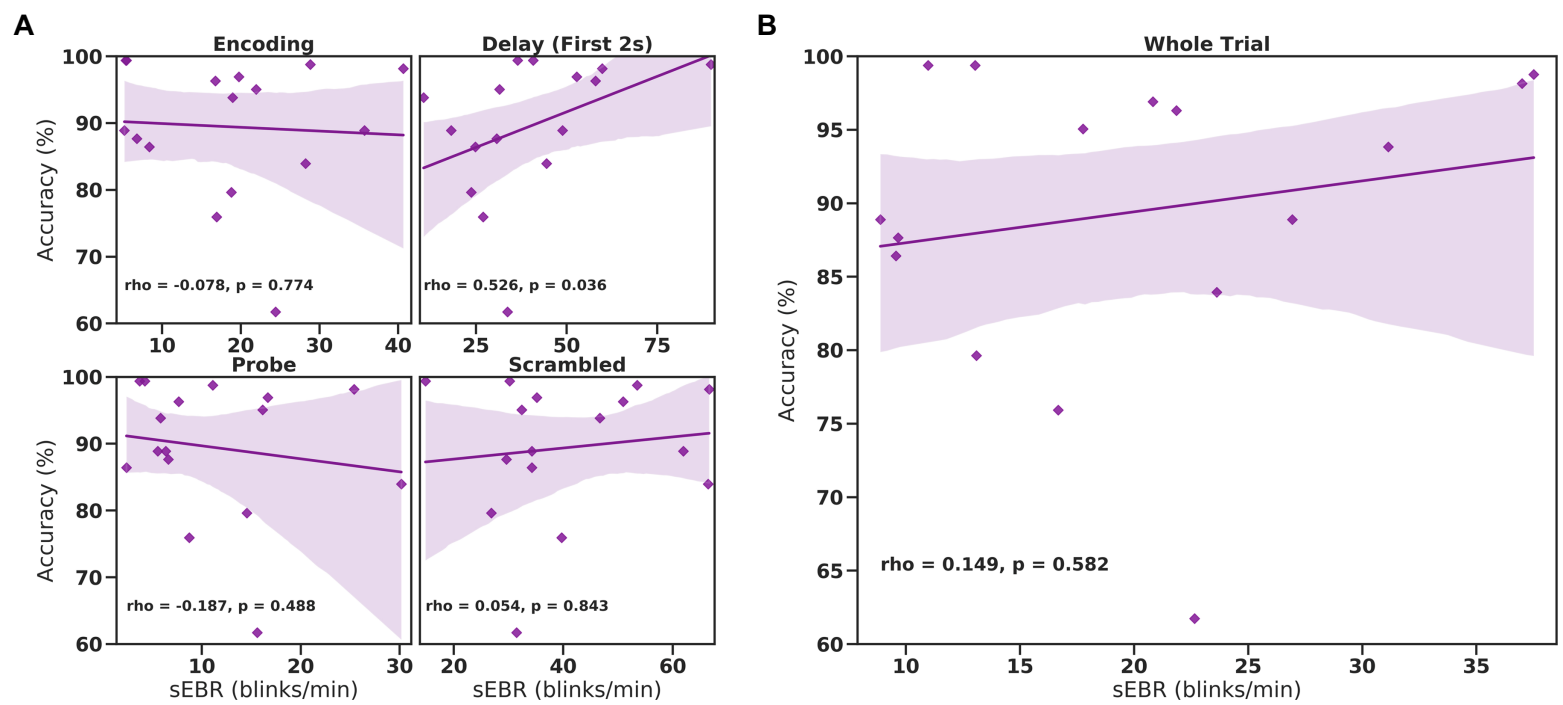
Correlation between sEBR during different phases of the task and task accuracy in Experiment 1. Correlation plots show sEBR (blinks/min) on the x-axis and task accuracy on the y-axis. **(A)** These four plots are encoding (top left), the first 2 s of the delay (top right), probe (bottom left), and scrambled (bottom right) periods. The delay period shows a positive correlation (*p* = 0.036 but not significant after multiple comparisons correction) between task accuracy and sEBR during the first 2 s of the delay period. **(B)** This plot represents the relationship between sEBR during the whole trial and task accuracy. Fitted line represents linear regression model fit. Shaded region depicts 95% confidence interval.

**Figure 3:**
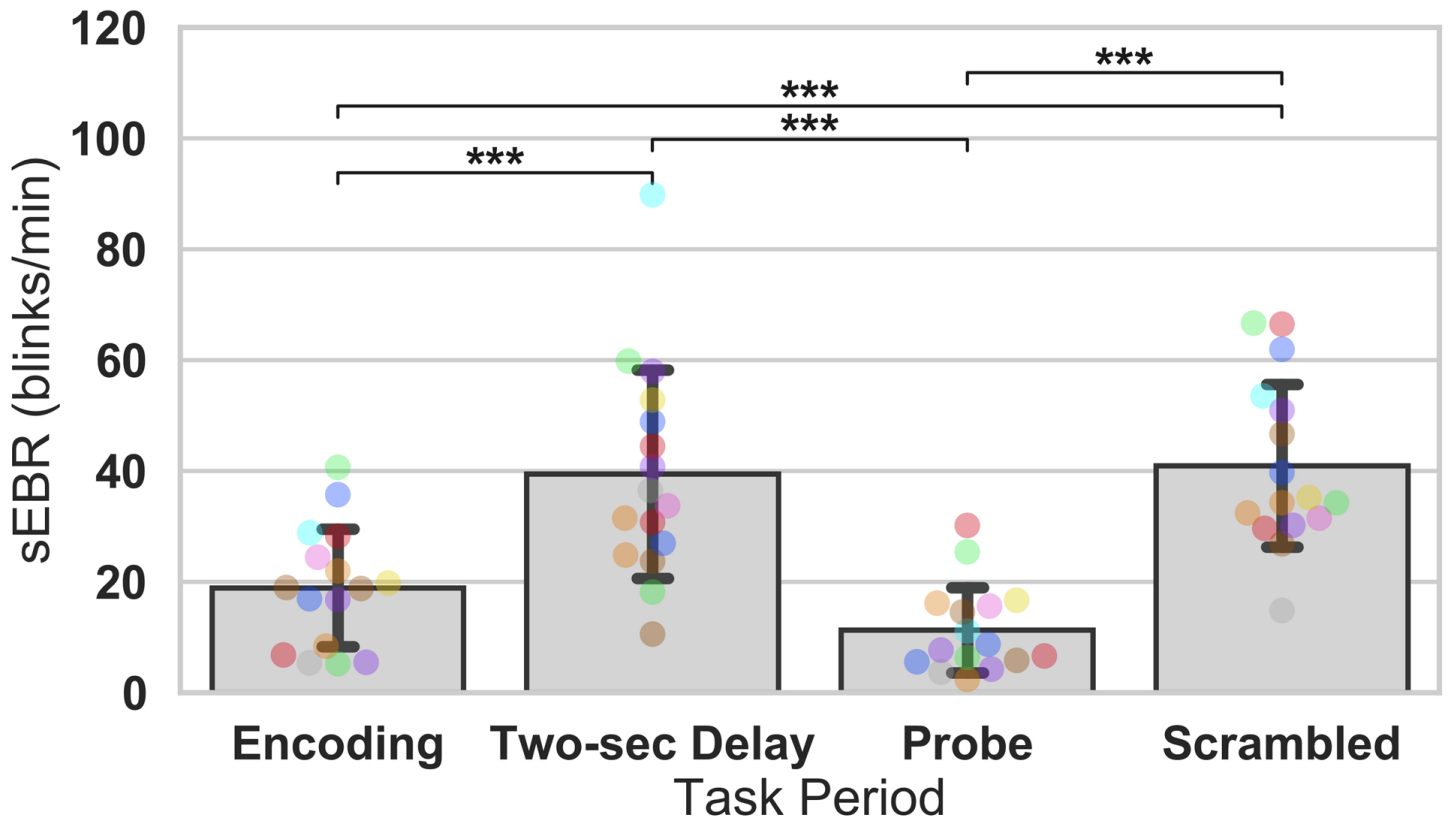
ANOVA test of sEBR across task periods in Experiment 1. Bar plots show task period on the x-axis and sEBR (blinks/min) on the y-axis. Delay period sEBR was significantly greater than Encoding and Probe sEBR. Scrambled sEBR was also significantly greater than Encoding and Probe sEBR. Error bars depict 95% confidence interval. Each colored circle represents an individual participant; some colors may be presented twice in one bar due to limited primary colors available for display. Values of *p* were adjusted for comparing a family of 4. ^***^p < 0.001.

**Figure 4:**
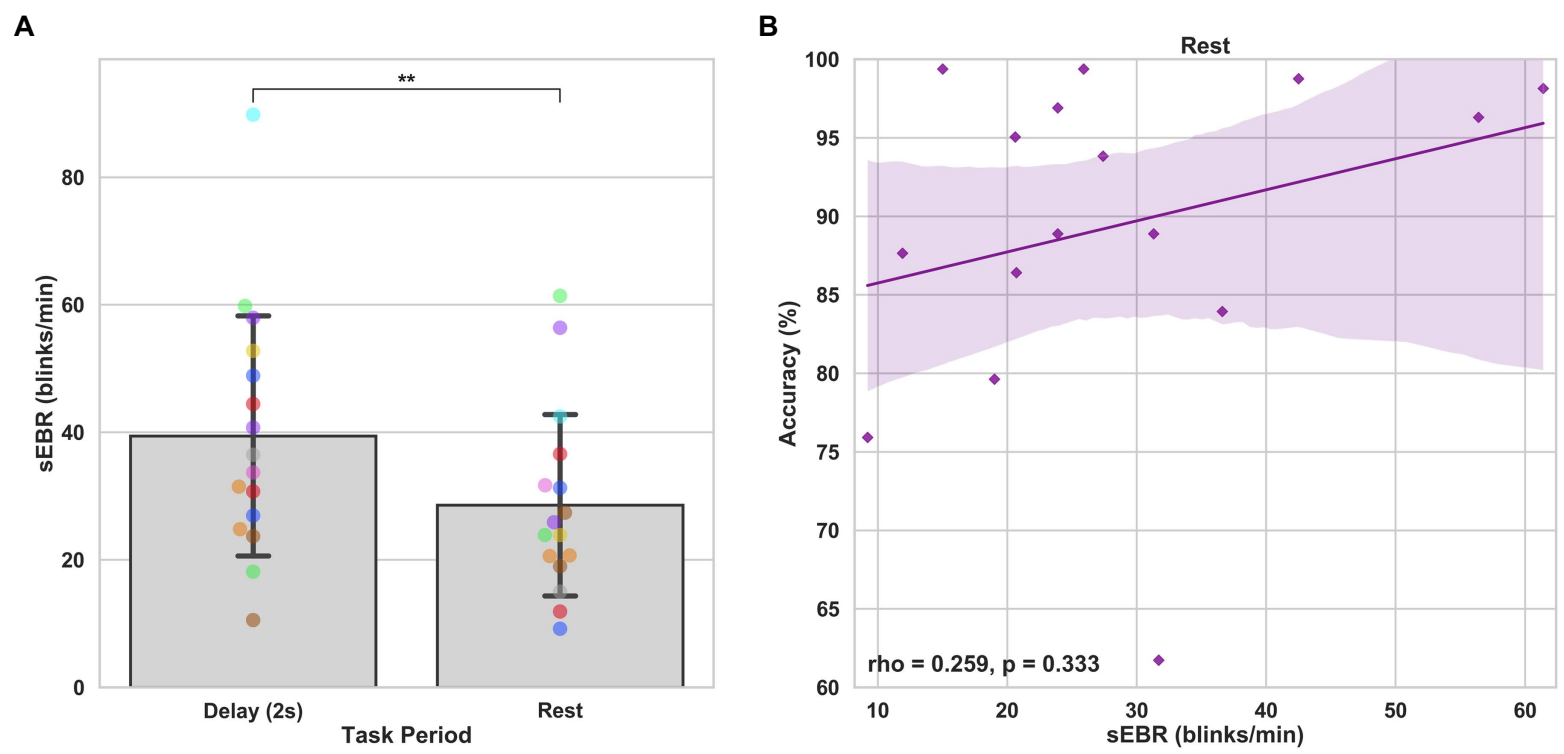
Paired *T* tests between Delay period sEBR and Rest sEBR and correlation between Rest sEBR and task accuracy in Experiment 1. **(A)** Bar plots show task period on the x-axis and sEBR on the y-axis. Delay period sEBR was significantly higher than Rest sEBR. Error bars depict 95% confidence interval. **(B)** Correlation plot of sEBR during the Rest period on the x-axis and task accuracy on the y-axis. Fitted line represents linear regression model fit. Shaded region depicts 95% confidence interval. ^**^p < 0.01.

In Experiment 1, we examined the relationship between sEBR and WM task performance while the duration of the WM delay period interval was varied. The first 2 s of all Delay periods were used in the analysis. First, because of the previously reported non-linear relationship between DA and WM task performance (Cools and D’Esposito, 2011), Spearman’s rho correlation coefficient (*r*_s_) was used to analyze the relationship between sEBR and task accuracy. After performing Bonferroni multiple comparisons correction on values of *p*, we found no significant relationship between sEBR during the phases of the task and task accuracy (Figure 2A). However, there was a strong positive correlation between sEBR during the Delay period and task accuracy (*r*_s_ = 0.526, *p* = 0.036; Figure 2A). We then examined the correlation between sEBR during the whole trial period and task accuracy to make sure that this relationship was not driving the relationship between sEBR during the Delay and task accuracy. There was no significant relationship between sEBR during the whole trial and task accuracy (*r*_s_ = 0.149, *p* = 0.582; Figure 2B). Descriptive statistics and reliability measures for Experiment 1 are presented in Table 1. Second, we computed a repeated measures ANOVA test to compare participants’ sEBR across the task phases. A Mauchly’s test of sphericity was first computed to check the assumption of sphericity in the data and was found to be significant (*p* = 0.012). Greenhouse-Geisser and Huynh-Feldt ε values were smaller than 0.75 so a Greenhouse-Geisser correction was performed. There were significant differences in sEBR between group means [*F* (1.948, 29.213) = 33.196, *p* < 0.001]. A *post-hoc* test using the Holm correction revealed that sEBR was significantly lower during Encoding (18.9 ± 11.0 sEBR, *p* < 0.001) and Probe (11.3 ± 8.0 sEBR, *p* < 0.001) periods compared to the Delay period (39.4 ± 19.5 sEBR; Figure 3). There was no significant difference in sEBR between the Delay and Scrambled period (*p =* 0.682). sEBR was also significantly lower during Encoding (18.9 ± 11.0 sEBR, *p* < 0.001) and Probe (11.3 ± 8.0 sEBR, *p* < 0.001) periods compared to the Scrambled period (40.9 ± 15.2 sEBR; Figure 3). Finally, we investigated the difference between phasic sEBR during the Delay period and tonic sEBR during the Rest period. We performed a paired samples *T* test to compare sEBR during the Delay and during Rest. We observed sEBR to be significantly higher during the Delay period (39.4 ± 19.5 sEBR) compared to the Rest period (28.6 ± 14.7 sEBR), *t*(15) = 2.885, *p* = 0.0011 (Figure 4A). We then investigated the correlation between tonic sEBR during the Rest period and task accuracy. There was no significant correlation between sEBR during the Rest period and task accuracy (*r*_s_ = 0.259, *p* = 0.333; Figure 4B).

**Table 1:** Descriptive statistics and split-half reliability for Experiment 1.

Variable	*n*	*M*	*SD*	Skewness	Kurtosis	Split-half coefficient
Task Accuracy (%)	16	89.43	10.34	−1.41	2.15	0.944
Whole Trial sEBR	16	20.07	9.40	0.62	−0.60	0.906
Encoding sEBR	16	18.89	10.95	0.39	−0.54	0.965
Delay sEBR	16	39.44	19.45	1.06	1.73	0.974
Probe sEBR	16	11.29	7.97	1.17	0.83	0.903
Scrambled sEBR	16	40.94	15.18	0.45	−0.62	0.961
Rest sEBR	16	28.59	14.68	1.05	0.73	

*M and SD represent mean and standard deviation, respectively. Split-half reliability based on 1,000 bootstrap replicates*.

### Experiment 2

**Figure 5:**
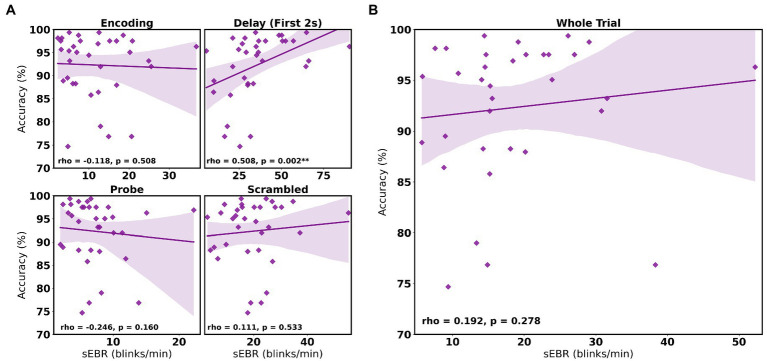
Correlation between sEBR during different phases of the task and task accuracy in Experiment 2. Correlation plots show sEBR on the x-axis and task accuracy on the y-axis. **(A)** These four plots are encoding (top left), the first 2 s of the delay (top right), probe (bottom left), and scrambled (bottom right) periods. The delay period shows a strong positive correlation (*p* = 0.002, significant after a multiple comparison correction) between task accuracy and sEBR during the first 2 s of the delay period. **(B)** This plot represents the relationship between sEBR during the whole trial and task accuracy. Fitted line represents linear regression model fit. Shaded region depicts 95% confidence interval. Values of *p* for **(A)** after Bonferroni correction: ^**^p < 0.0025.

**Figure 6:**
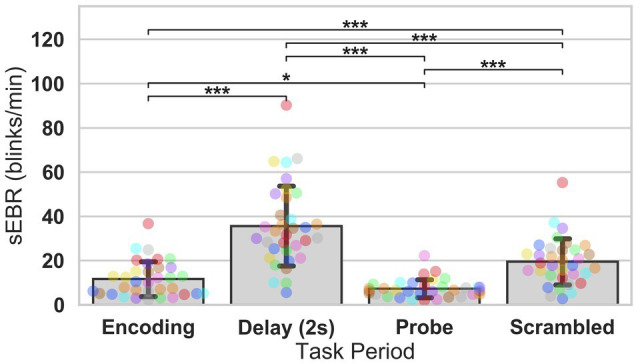
ANOVA test of sEBR across task periods in Experiment 2. Bar plots show task period on the x-axis and sEBR on the y-axis. Delay period sEBR was significantly greater than Encoding, Probe, and Scrambled sEBR. Scrambled sEBR was also significantly greater than Encoding and Probe sEBR. Encoding sEBR was significantly greater than Probe sEBR. Error bars depict 95% confidence interval. Each colored circle represents an individual participant; some colors may be presented twice in one bar due to limited primary colors available for display. Values of *p* were adjusted for comparing a family of 4. ^*^p < 0.05, and ^***^p < 0.001.

**Figure 7:**
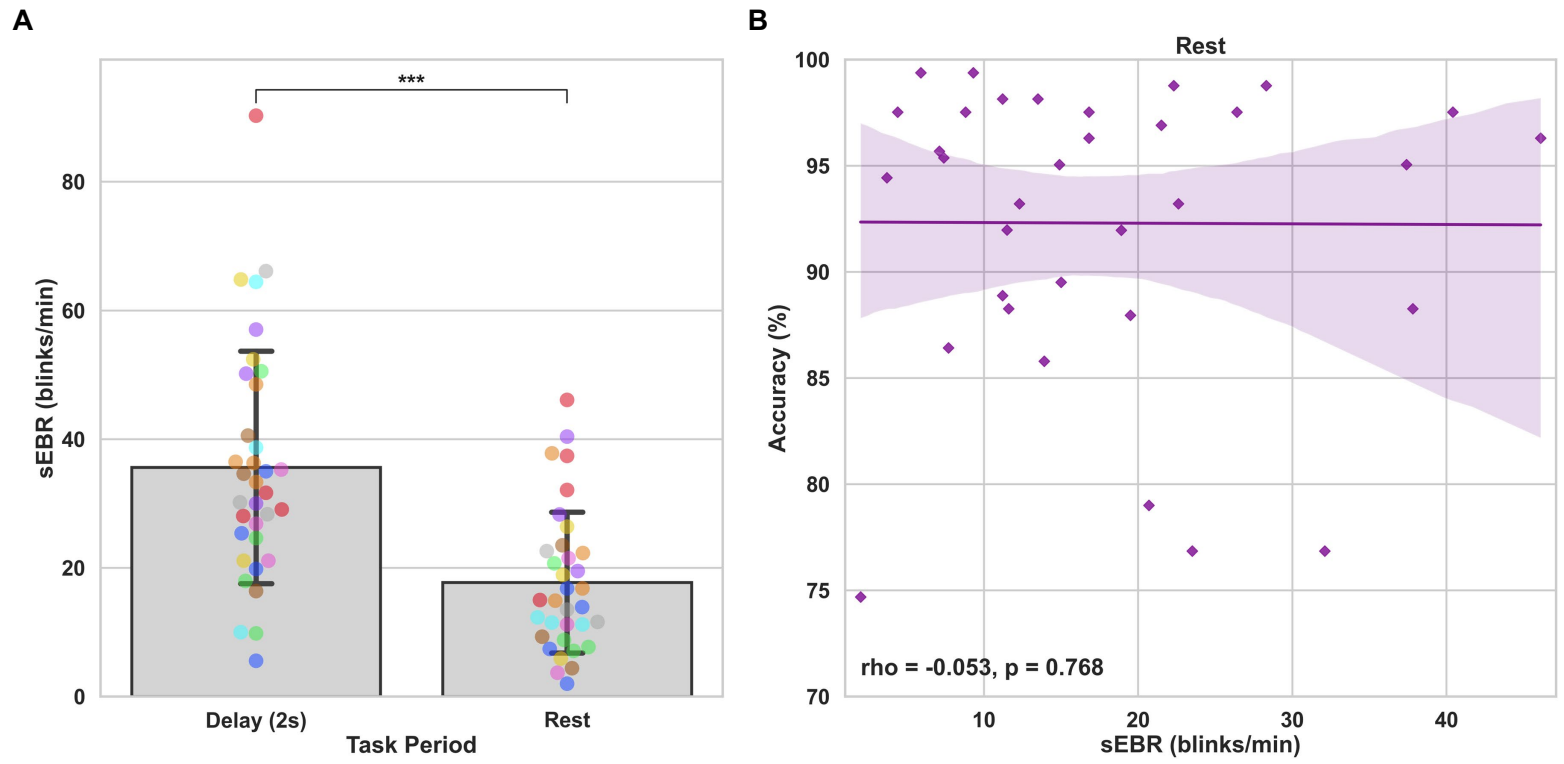
Paired T tests between Delay period sEBR and Rest sEBR and correlation between Rest sEBR and task accuracy in Experiment 2. **(A)** Bar plots show task period on the x-axis and sEBR on the y-axis. Delay period sEBR was significantly higher than Rest sEBR. Error bars depict 95% confidence interval. **(B)** Correlation plot of sEBR during the Rest period on the x-axis and task accuracy on the y-axis. Fitted line represents linear regression model fit. Shaded region depicts 95% confidence interval. ^***^p < 0.001.

Experiment 2 included a larger sample of subjects with a task design identical to Experiment 1. First, we examined the relationship between sEBR during each WM task phase and task accuracy. After performing Bonferroni correction on values of *p*, we found that sEBR during the WM delay period was correlated positively with task performance (*r*_s_ = 0.508, *p* = 0.002), with no significant relationships observed between sEBR in other task periods and task performance (Figure 5A). We then examined the relationship between sEBR during the whole trial and task accuracy to make sure that the significant relationship between Delay sEBR and task accuracy was not driven by sEBR during the whole trial. We found no significant relationship between whole trial sEBR and task accuracy (*r*_s_ = 0.192, *p* = 0.278; Figure 5B). Descriptive statistics and reliability measures for Experiment 2 are presented in Table 2. We then repeated the same analysis of comparing sEBR across the task phases by computing a repeated measures ANOVA test. A Mauchly’s test of sphericity was first computed to check the assumption of sphericity in the data and was found to be significant (*p* < 0.001). Greenhouse-Geisser and the Huynh-Feldt ε values were smaller than 0.75 so a Greenhouse-Geisser correction was performed. There were significant differences in sEBR between group means [*F* (1.578,52.058) = 66.958, *p* < 0.001]. A *post-hoc* test using the Holm correction revealed that sEBR was significantly lower during Encoding (11.6 ± 8.0 sEBR, *p* < 0.001), Probe (7.3 ± 4.1 sEBR, *p* < 0.001), and Scrambled (19.5 ± 10.6 sEBR, *p* < 0.001) periods compared to the Delay period (35.6 ± 18.3 sEBR; Figure 6). sEBR was also significantly lower during the Encoding (11.6 ± 8.0 sEBR, *p* < 0.001) and Probe (7.3 ± 4.1 sEBR, *p* < 0.001), periods compared to the Scrambled period (19.5 ± 10.6 sEBR; Figure 6). Additionally, sEBR was significantly lower during the Probe (7.3 ± 4.1 sEBR, *p* = 0.047), period compared to the Encoding period (11.6 ± 8.0 sEBR; Figure 6). We then investigated the difference between sEBR during the Delay period and sEBR during the Rest period. We performed a paired samples T test to compare sEBR during the Delay and during Rest. We observed sEBR to be significantly higher during the Delay period (35.6 ± 18.3 sEBR) compared to the Rest period (17.7 ± 11.1 sEBR), *t*(33) = 6.005, *p* < 0.001 (Figure 7A). We then investigated the correlation between tonic sEBR during the Rest period and task accuracy. There was no significant correlation between sEBR during the Rest period and task performance (*r*_s_ = −0.053, *p* = 0.768; Figure 7B).

**Table 2:** Descriptive statistics and split-half reliability for Experiment 2.

Variable	*n*	*M*	*SD*	Skewness	Kurtosis	Split-half coefficient
Task Accuracy (%)	34	92.30	6.956	−1.266	0.765	0.678
Whole Trial sEBR	34	18.486	9.843	1.453	3.023	0.992
Encoding sEBR	34	11.63	7.979	1.153	1.433	0.971
Delay sEBR	34	35.605	18.336	0.918	1.127	0.987
Probe sEBR	34	7.298	4.112	1.67	4.208	0.992
Scrambled sEBR	34	19.474	10.582	1.092	2.72	0.992
Rest sEBR	34	17.721	11.121	0.911	0.256	

*M and SD represent mean and standard deviation, respectively. Split-half reliability based on 1,000 bootstrap replicates*.

### Polynomial Regression Model

**Figure 8:**
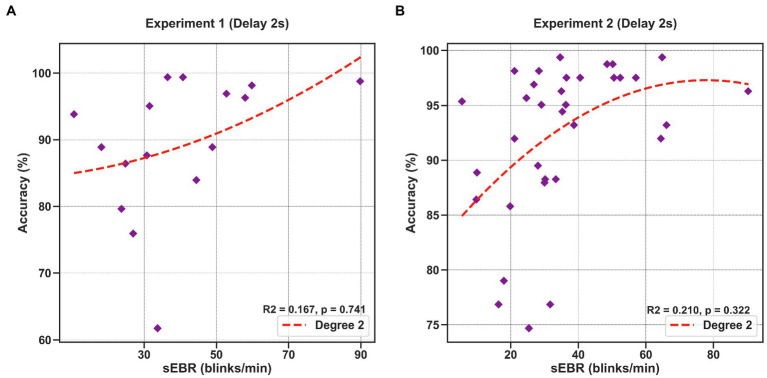
Polynomial regression model between task accuracy and sEBR during the first 2 s of the delay for Experiment 1 and Experiment 2. Regression plots show sEBR during the first 2 s of the Delay on the x-axis and task accuracy on the y-axis. **(A)** Polynomial regression model fitted on sEBR during the Delay and task accuracy in Experiment 1. **(B)** Polynomial regression model fitted on sEBR during the Delay and task accuracy in Experiment 2. Fitted red line represents polynomial regression model fit. The relationship between sEBR and WM performance appears to be non-linear and explains about 20% of the variance in Experiment 2 but does not reach significance.

To investigate whether sEBR during the Delay varies non-linearly with task performance, we computed a quadratic polynomial regression model between sEBR during the Delay period of Experiment 1 and Experiment 2 and task accuracy. There was no significant polynomial regression relationship found between task accuracy and the first 2 s of the Delay in Experiment 1 (*β* = 0.324, *p* = 0.741) nor between task accuracy and the first 2 s of the Delay in Experiment 2 (*β* = −0.568, *p* = 0.322; Figure 8).

### Reliability and Statistical Power

Reliability of each measure was computed using a split-half analysis procedure. Each measure was divided into two subsets, at random, by trial and recomputed. Correlation coefficients were then calculated on both subsets across participants. The split-half reliability correlation coefficient was permuted 1,000 times and the average of the correlations is reported for each independent measure. Before averaging, all correlations were Fisher Z-transformed and then transformed back after averaging. In order to correct for underestimation resulting from splitting the number of observations in half, the Spearman-Brown correction was applied (Parsons et al., 2019). Reliability was not computed for sEBR during the rest period since it contained no trials. Descriptive statistics and split-half reliability measures are summarized in Tables 1 and 2 for experiment 1 and experiment 2, respectively. Correlations between sEBR and performance with associated values of *p* and confidence intervals are summarized in Tables 3 and 4 for experiment 1 and experiment 2, respectively. Post-hoc statistical power calculations using as the effect size the correlation between sEBR during the Delay period and task accuracy showed inadequate power for experiment 1 (*N* = 16, 1-β error probability = 0.53, critical *r* = 0.49). Power for experiment 2 was good (*N* = 34, 1-β error probability = 0.87, critical *r* = 0.33). Given that experiments 1 and 2 utilized different methods of quantifying blinks (camera based vs. EOG) but a similar task design, the two sample sizes were combined with very good power obtained (*N* = 50, 1-β error probability = 0.96, critical *r* = 0.27).

**Table 3:** Correlations between sEBR and performance for Experiment 1.

Variable	Spearman’s rho	Value of *p*	95% CI
Encoding sEBR	−0.078	0.774	[−0.569, 0.490]
Delay sEBR	0.526	0.036	[0.121, 0.783]
Probe sEBR	−0.187	0.488	[−0.699, 0.401]
Scrambled sEBR	0.054	0.843	[−0.477, 0.623]
Rest sEBR	0.259	0.333	[−0.285, 0.740]
Whole trial sEBR	0.149	0.582	[−0.369, 0.676]

*CI, confidence intervals. CIs based on 1,000 bootstrap replicates*.

**Table 4:** Correlations between sEBR and performance for Experiment 2.

Variable	Spearman’s rho	Value of *p*	Confidence Intervals
Encoding sEBR	−0.118	0.508	[−0.418, 0.224]
Delay sEBR	0.508	0.002	[0.213, 0.699]
Probe sEBR	−0.246	0.16	[−0.527, 0.082]
Scrambled sEBR	0.111	0.533	[−0.228, 0.423]
Rest sEBR	−0.053	0.768	[−0.400, 0.310]
Whole trial sEBR	0.192	0.278	[−0.148, 506]

*CI, confidence intervals. CIs based on 1,000 bootstrap replicates*.

## Discussion

In the present study, we investigated the temporal fluctuations in sEBR across a WM paradigm and its relation to WM task accuracy in two experiments, inside and outside an MRI scanner, and using two methods of collecting sEBR. Using the same Sternberg working memory paradigm, we observed a strong positive relationship between sEBR and task performance only during the WM task delay in both experiments. We also found a significant difference in sEBR between task phases and a difference between Delay period sEBR and baseline sEBR.

Our first hypothesis was that phasic sEBR during the Delay period of the WM task would be positively correlated with task accuracy and that we would also observe a non-linear relationship where high and low sEBR would be predictive of low performance. We observed a strong positive correlation between sEBR during the Delay period in both Experiment 1 and Experiment 2 with task accuracy. However, only in Experiment 2 was this relationship significant. We believe that the lack of significance in Experiment 1 is due to the smaller sample size and thus lack of power, which our formal post-hoc power analyses confirmed. While the sample size in Experiment 2 was also small, we observed a similar correlation and reliability statistic as well as higher power while recording sEBR using a different method (electrooculogram instead of camera-based eye-tracking hardware). Nevertheless, a correlation between sEBR and task performance of approximately 0.50 is a very high correlation in psychology, especially between a behavioral measure and a physiological measure (Gignac and Szodorai, 2016). The replication of a similar correlation between Delay period sEBR and WM performance across two separate experiments strengthens our findings but with the confidence interval being so wide with the relatively small sample size one cannot be certain the true correlation is so large. Previous research has found that correlations begin to stabilize at even larger sample sizes (Schönbrodt and Perugini, 2013). Thus, future studies should include an additional experiment with high statistical power to replicate these findings and to determine whether the observed effect stabilizes with even larger sample sizes.

If we interpret sEBR as an indirect measure of striatal DA activity, as other studies have postulated, we can speculate that higher sEBR during the WM delay was correlated with task accuracy due to DA regulating the maintenance and updating of representations in WM (Westbrook and Braver, 2016). The other results support this idea since no other task period was significantly correlated with task accuracy. Many studies that have investigated the relationship between sEBR and cognitive functions have used baseline levels of sEBR taken before or after tasks in their analysis (Tharp and Pickering, 2011; Zhang et al., 2015; Unsworth et al., 2019b). However, we show that while the WM task Delay period sEBR was correlated positively with task accuracy, baseline levels of sEBR were not. Our results highlight the importance of examining phasic and tonic sEBR when investigating the relationships between sEBR and other cognitive functions. The results also highlight that blinking may be an important component of working memory function; however, future studies, including within-subject analyses using larger number of trials, are needed to understand the role of blinking during WM maintenance. Additionally, future studies should investigate whether higher blink rates during the WM delay lead to a correct response. Since task difficulty was not controlled for in this study, participants’ task performance in both experiments was relatively high (see Tables 1 and 2). These limitations make our current dataset incapable of investigating these questions.

We also investigated the proposed “Inverted U-shape” relationship between DA and WM performance by computing a polynomial regression model on sEBR during the delay and task accuracy (Cools and D’Esposito, 2011). Though the model showed a non-linear trend in Experiment 2, the model was not significant. We believe that failure to achieve non-linear model significance was due to lack of extreme (sub- and supraoptimal) sEBRs observed in the pool of participants, which are typically found in clinical populations (e.g., with Schizophrenia; Adamson, 1995; Swarztrauber and Fujikawa, 1998). Future studies should investigate sEBR with healthy subjects and with subjects that have been observed to have extreme sEBR in order to have a wider variety of sEBRs and to better understand its connection with DA. Additionally, other methods of DA measures could be used to investigate DA during the delay period, such as correlations with neuromelanin-sensitive MRI, which can detect neuromelanin, a product of dopamine metabolism (Cassidy et al., 2019).

Our second hypothesis was that we would see significant differences in sEBR across the WM task as well as between sEBR during Rest and during the Delay period. We found sEBR to be the lowest during periods like Encoding and Probe in both Experiments, while sEBR during the Delay was the highest. Our results support previous findings which found task-related modulation of sEBR (Siegle et al., 2008; Oh et al., 2012). Prior work has found sEBR to be lower during tasks that require visual attention (Fukuda et al., 2005; Oh et al., 2012). This would explain the lower sEBR’s observed during the Encoding period when participants are encoding information into WM and during the Probe period where participants are retrieving information from WM. We also found that sEBR was the highest during the Delay period when participants were maintaining information in WM. This was also demonstrated in a different study which investigated sEBR during an A-not-B WM task where infants had to search for a hidden toy by making an eye movement to one of two locations (Bacher et al., 2017). Higher sEBR during the WM delay could be due to DA regulating the maintenance and updating of representations in WM (Westbrook and Braver, 2016), but this remains speculation until further studies directly measure dopaminergic activity during task performance. Our results further support this interpretation since Delay period sEBR was significantly higher than baseline sEBR during the Rest period. Lower sEBR during the Rest period could be explained since there is no need to update or maintain WM during this period.

To conclude, we investigated temporal changes of sEBR during different phases of a WM task and its relation to WM performance. We observed a significant positive correlation between sEBR and WM task performance during the Delay period, but not during the other phases of the task. Additionally, we found evidence for an association of sEBR during both stimulus encoding and WM probe retrieval, potential reflecting visual attention. To the best of our knowledge, this is the first study to investigate phasic and tonic sEBR during different phases of a WM task using complex visual scenes. Future studies should continue to investigate sEBRs in relation to direct measures of cortical (especially PFC) and subcortical dopamine and assess linear and non-linear relationships to task performance in healthy and clinical populations (e.g., Schizophrenia and Parkinson’s disease).

## Data Availability Statement

The raw data supporting the conclusions of this article will be made available by the authors, without undue reservation.

## Ethics Statement

The studies involving human participants were reviewed and approved by the Institutional Review Board of the City University of New York Human Research Protection Program (CUNY HRPP IRB). The participants provided their written informed consent to participate in this study.

## Author Contributions

TE designed the study. JO, CR, and BG performed the research. JO analyzed and interpreted the data, prepared the figures, and wrote the final manuscript. CR, BG, and TE edited and reviewed the manuscript. All authors contributed to the article and approved the submitted version.

## Funding

Research reported in this publication was supported by the National Institute of Mental Health of the National Institutes of Health under Award Number R56MH116007 (TE).

## Conflict of Interest

The authors declare that the research was conducted in the absence of any commercial or financial relationships that could be construed as a potential conflict of interest.

## Publisher’s Note

All claims expressed in this article are solely those of the authors and do not necessarily represent those of their affiliated organizations, or those of the publisher, the editors and the reviewers. Any product that may be evaluated in this article, or claim that may be made by its manufacturer, is not guaranteed or endorsed by the publisher.
